# Full L_1_-regularized Traction Force Microscopy over whole cells

**DOI:** 10.1186/s12859-017-1771-0

**Published:** 2017-08-10

**Authors:** Alejandro Suñé-Auñón, Alvaro Jorge-Peñas, Rocío Aguilar-Cuenca, Miguel Vicente-Manzanares, Hans Van Oosterwyck, Arrate Muñoz-Barrutia

**Affiliations:** 1 Bioengineering and Aerospace Engineering Department, Universidad Carlos III de Madrid, Leganés, Spain; 2 Instituto de Investigación Sanitaria Gregorio Marañón, 28911 Madrid, Spain; 3 Biomechanics Section, Department of Mechanical Engineering, KU Leuven, 3001 Leuven, Belgium; 4 Instituto de Investigación Sanitaria-Hospital Universitario de la Princesa, Universidad Autónoma de Madrid, School of Medicine, 28006 Madrid, Spain; 5 Prometheus, Division of Skeletal Tissue Engineering, KU Leuven, Leuven, Belgium

**Keywords:** Traction Force Microscopy, Spatial domain, Regularization, Spatial resolution

## Abstract

**Background:**

Traction Force Microscopy (TFM) is a widespread technique to estimate the tractions that cells exert on the surrounding substrate. To recover the tractions, it is necessary to solve an inverse problem, which is ill-posed and needs regularization to make the solution stable. The typical regularization scheme is given by the minimization of a cost functional, which is divided in two terms: the error present in the data or data fidelity term; and the regularization or penalty term. The classical approach is to use zero-order Tikhonov or L_2_-regularization, which uses the L_2_-norm for both terms in the cost function. Recently, some studies have demonstrated an improved performance using L_1_-regularization (L_1_-norm in the penalty term) related to an increase in the spatial resolution and sensitivity of the recovered traction field. In this manuscript, we present a comparison between the previous two regularization schemes (relying in the L_2_-norm for the data fidelity term) and the full L_1_-regularization (using the L_1_-norm for both terms in the cost function) for synthetic and real data.

**Results:**

Our results reveal that L_1_-regularizations give an improved spatial resolution (more important for full L_1_-regularization) and a reduction in the background noise with respect to the classical zero-order Tikhonov regularization. In addition, we present an approximation, which makes feasible the recovery of cellular tractions over whole cells on typical full-size microscope images when working in the spatial domain.

**Conclusions:**

The proposed full L_1_-regularization improves the sensitivity to recover small stress footprints. Moreover, the proposed method has been validated to work on full-field microscopy images of real cells, what certainly demonstrates it is a promising tool for biological applications.

**Electronic supplementary material:**

The online version of this article (doi:10.1186/s12859-017-1771-0) contains supplementary material, which is available to authorized users.

## Background

Tissue remodeling implies the reorganization of the extracellular matrix (ECM), which is driven by the conversion of intracellular-generated mechanical forces into extracellular traction, which reorganizes the ECM fibers. This is a crucial process during regeneration (e.g., in wound healing) but it is equally important in pathologic scenarios (e.g., in inflammation and/or cancer). In fact, some of the pathological changes associated to these diseases are due to the altered ability of cancer, or inflammatory cells, to exert abnormal traction on their microenvironment [1]. Thus, the precise quantification of the traction exerted by cells (and groups of cells) is key to understand the remodeling processes that occur during these biologically relevant events, including events on the cellular scale (e.g., cell migration and division) as well as at a subcellular scale (e.g., signal transduction events). Cells convert intracellular-generated forces into extracellular-applied tractions and transmit them to the microenvironment through micron-sized protein accumulations termed focal adhesions (FA) [2–8].

Traction Force Microscopy (TFM) is a technique that estimates the tractions exerted by adherent cells placed on top of a flexible hydrogel that mimics the mechanical properties of the ECM [9].

**Fig. 1:**
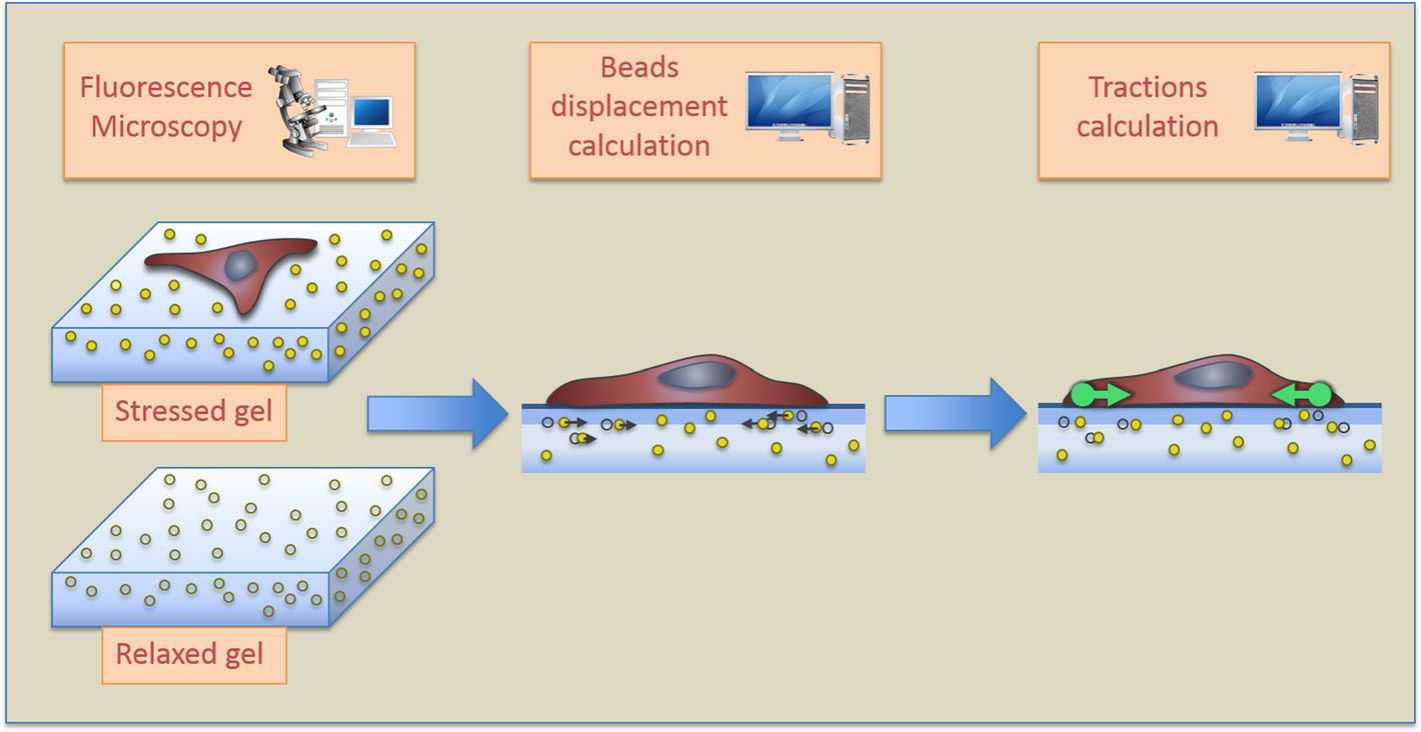
Schematic representation of a Traction Force Microscopy experiment and the generic blocks of the traction recovery algorithms

In a typical TFM experiment, a large number of fluorescent beads are randomly mixed inside the hydrogel. The fluorescent beads are displaced as the cells exert tractions on the substratum and act as fiduciary markers enabling the transformation of motion into force (see Fig. 1). Experimentally, fluorescence microscopy is used to acquire images of the beads before (stressed hydrogel) and after (relaxed hydrogel) suppressing cell tractions, either by drug-induced relaxation of cells, or directly via cell detachment or lysis. The images of the stressed and relaxed hydrogel are then compared to track the motion of the embedded beads and obtain the matrix displacement field. This, together with a mechanical model of the hydrogel based on its height and its elasticity (Young’s modulus) allows estimating the cellular tractions using computational methods.

The displacement field of the beads can be obtained using different image processing methods. Two classical approaches used in TFM experiments include block-wise image correlation computation (Particle Image Velocimetry) [10]; and individual motion tracking of each bead (Particle Tracking Velocimetry) [11]. One of the novel aspects of the present study is that we use a non-rigid image registration approach, which provides very good accuracy with a reasonable computation time and without a loss in spatial resolution compared to previous methods [12].

Recovering cellular tractions (causes) from matrix displacements (observable effects) implies solving an ill-posed inverse problem. Consequently, the solution is very sensitive to the errors committed in the calculation of the displacement field.

To constraint the solution and prevent the estimated tractions from over-fitting the noise present in the matrix displacements, it is common to regularize the traction reconstruction process, including a penalty term in the norm of the tractions [13]. Historically, L_2_-norm regularization, also known as Tikhonov regularization [14], is the most widely used scheme in TFM, as it allows an efficient traction recovery in the frequency domain [15]. However, recent studies showed that the use of the L_1_-norm in the imposed penalty term yields improved results in terms of spatial resolution, estimation of the traction magnitude and background noise reduction, giving sparser traction fields as is expected in TFM experiments [16–18]. For example, L_1_-regularization was introduced to study the focal adhesions maturation, which was possible by the huge improvement obtained in the spatial resolution [16]. Using a similar approach Bohr et al. in [17], demonstrated that traction fields with high spatial resolution could be computed from displacement fields with low resolution.

A major issue with L_1_-regularization is that it requires solving the problem in the spatial domain instead of the frequency domain, which greatly increases the computational demand. Therefore, its application has been limited to either reduced regions of interest with full-resolution displacements, or full field of views with low-resolution displacements.

To overcome this limitation, in this study, we introduce an approximation that reduces the computational cost of recovering the tractions in the spatial domain, thus, providing the means to work with both full-field microscopy images (covering whole cells) and full-resolution displacements. In addition, we propose the use of full L_1_-regularization in TFM, which minimizes the L_1_-norm of both the penalty (i.e., recovered tractions) and the data fidelity terms (i.e., difference between the estimated displacements and the ones obtained from the given recovered tractions). Here, the performance of the proposed scheme satisfactorily compares to the classical approach (regularization minimizing the L_2_-norm for both the penalty and the data fidelity terms) and the simple L_1_-regularization (constraining the L_1_-norm of the penalty term) with synthetic and real data.

In the following sections, we present the foundation of 2D TFM traction recovery in the spatial domain and the method implemented to compute the traction forces for full-size microscopy images, followed by the evaluation of synthetic data and experiments with live cells, respectively. Results section contains the experimental results, including a qualitative and quantitative comparison between the different regularization schemes, followed by a discussion on the limitations of the present approach and future perspectives on the application of this technique.

## Methods

### Regularized Traction Force Microscopy

In this work, we consider a bidimensional (2D) TFM scenario in which cells are cultured on the top of a controlled thickness hydrogel. One assumption is that cells only exert in-plane (shear) forces on the surface of the hydrogel. In this case, hydrogel deformation is obtained as the solution of the elasticity problem:

1 $$ {u}_j\left(\boldsymbol{x}\right)={\sum}_i\int {g}_{ji}\left(\boldsymbol{x},{\boldsymbol{x}}^{'}\right)\ {t}_i\left({\boldsymbol{x}}^{'}\right)\ d{\boldsymbol{x}}^{'} $$

where the *j*-th component (*j* ∈ {*x, y*}) of the displacement field **u** at spatial location **x** is related to the tractions **t** exerted along the *¡*-th Cartesian direction (*¡* ∈ {*x, y*}) at the location **x**′ by the Green’s function of the hydrogel g(**x,x**′).

The Green’s function models the elastic deformation of the hydrogel in response to a point force. It is usually given by the analytical Boussinesq solution in 2D TFM, where the hydrogel can be approximated by a homogeneous, isotropic, elastic and semi-infinite medium. Under these assumptions, the Green function can be considered linear shift-invariant [19], which allows transforming Eq. 1 in a summation of convolutions:

2 $$ {u}_j\left(\boldsymbol{x}\right)={\sum}_i\int {g}_{ji}\left(\boldsymbol{x}-{\boldsymbol{x}}^{'}\right)\ {t}_i\left({\boldsymbol{x}}^{'}\right)\ d{\boldsymbol{x}}^{'}={\sum}_i{g}_{ji}\left(\boldsymbol{x}\right)\bigotimes {t}_i\left(\boldsymbol{x}\right) $$

In practice, displacement and traction fields are only estimated at discrete locations of the space, and Eq. (2) can be rewritten in matrix form as:

3 $$ \mathbf{u}=\mathbf{K}\cdotp \mathbf{t} $$

where **u** = [*u*_*x*_(***x***_1_);  … ; *u*_*x*_(***x***_*N*_); *u*_*y*_(***x***_1_);  … ; *u*_*y*_(***x***_*N*_)] is a 2*N* × 1 column vector with *N* being the number of locations where displacements are measured, $\mathbf{t}=\left[{t}_x\left(\boldsymbol{x}{'}_1\right);\dots; \left({\boldsymbol{x}}_M^{'}\right);{t}_y\left({\boldsymbol{x}}_1^{'}\right);\dots; {t}_y\left(\boldsymbol{x}{'}_M\right)\right]$ is a 2*M* × 1 column vector with *M* being the number of locations where tractions will be estimated, and **K** is the 2*N* × 2*M* stiffness matrix given by:

4 $$ \mathbf{K}=\left[\begin{array}{cc}{\mathbf{G}}_{xx}& {\mathbf{G}}_{xy}\\ {}{\mathbf{G}}_{yx}& {\mathbf{G}}_{yy}\end{array}\right] $$

with

5 $$ {\mathbf{G}}_{ji}=\left[\begin{array}{ccc}{g}_{ji}\left(1,1\right)& \cdots & {g}_{ji}\left(1,M\right)\\ {}\vdots & \ddots & \vdots \\ {}{g}_{ji}\left(N,1\right)& \cdots & {g}_{ji}\left(N,M\right)\end{array}\right] $$

A trivial solution for the traction recovery is obtained by the direct inversion of Eq. 3. However, it implies the inversion of the stiffness matrix, which is ill-conditioned, and thus, it would amplify the errors present in the computed displacement field leading to unstable results. For this reason, a regularization scheme is commonly used to reach a stable solution. Then, the traction field recovery in the spatial domain is given by the minimization of the following cost functional:

6 $$ \widehat{\mathbf{t}}=\underset{\mathbf{t}}{\mathrm{argmin}}\left[\parallel \mathbf{Kt}-\mathbf{u}{\parallel}_q+\lambda \parallel \mathbf{t}{\parallel}_p\right] $$

where ‖·‖_*q*_ denotes the L_q_-norm, ∥**Kt** − **u**∥_*q*_ is the data fidelity term, ∥**t**∥_*p*_ is the imposed penalty and *λ* is a parameter that controls the amount of regularization applied.

#### L_2_-Regularization in the Fourier Domain

Following Parseval’s theorem, L_2_-regularization has an efficient, and widely used, implementation in Fourier domain [15], where the summation of convolutions in the spatial domain (Eq. 2) is transformed in a summation of products:

7 $$ {\overset{\sim }{u}}_j\left(\boldsymbol{k}\right)={\sum}_i{\overset{\sim }{g}}_{ji}\left(\boldsymbol{k}\right){\overset{\sim }{t}}_i\left(\boldsymbol{k}\right) $$

where $\overset{\sim }{u},\overset{\sim }{g},\overset{\sim }{t}$ are the Fourier transforms of *u* , *g* , *t* in Eq. 2 and ***k*** are the frequencies where these variables are evaluated.

Analogously to the spatial domain, the previous equation can be written in a matrix form:

8 $$ \overset{\sim }{\boldsymbol{u}}=\overset{\sim }{\boldsymbol{A}}\cdotp \overset{\sim }{\boldsymbol{t}} $$

where $\overset{\sim }{\boldsymbol{A}}$ contains all the values of ${\overset{\sim }{g}}_{ji}$ in Eq. 7.

Then, the L_2_-regularization can be formulated as the minimization of a cost functional, taking the L_2_-norm for both penalty and data fidelity terms:

9 $$ {\widehat{\boldsymbol{t}}}_{\boldsymbol{F},{\boldsymbol{L}}_2}=\underset{\mathbf{t}}{{\mathcal{F}}^{-1}\Big\{\mathrm{argmin}}\left[\parallel \overset{\sim }{\boldsymbol{A}}\overset{\sim }{\boldsymbol{t}}-\overset{\sim }{\boldsymbol{u}}{\parallel}_2+{\lambda}_{F,{L}_2}\parallel \overset{\sim }{\boldsymbol{t}}{\parallel}_2\right]\Big\} $$

being ℱ^−1^ the inverse Fourier transform.

The solution for the traction field is, thus, given by:

10 $$ {\widehat{\boldsymbol{t}}}_{\boldsymbol{F},{\boldsymbol{L}}_2}={\mathcal{F}}^{-1}\left\{{\left({\overset{\sim }{\boldsymbol{A}}}^{\ast}\overset{\sim }{\boldsymbol{A}}+{\lambda}_{F,{L}_2}\boldsymbol{I}\right)}^{-1}{\overset{\sim }{\boldsymbol{A}}}^{\ast}\overset{\sim }{\boldsymbol{u}}\right\} $$

where ${\overset{\sim }{\boldsymbol{A}}}^{\ast }$ is the complex conjugate of $\overset{\sim }{\boldsymbol{A}}$ **.**

#### L_2_-Regularization in the Spatial Domain

Zero order Tikhonov regularization can be also formulated in spatial domain, using the L_2_-norm for both terms in Eq. 6 (*p* = *q* = 2). Then, the minimization problem has an analytical solution given by [20]:

11 $$ {\widehat{\mathbf{t}}}_{{\mathrm{L}}_2}={\left({\mathbf{K}}^{\mathrm{T}}\mathbf{K}+{\lambda}_{L_2}\mathbf{I}\right)}^{-1}{\mathbf{K}}^{\mathrm{T}}\mathbf{u} $$

with **K** ^T^ being the transpose of the stiffness matrix.

#### L_1_-Regularization in the Spatial Domain

A better alternative is to use the L_1_-norm for the penalty term (*q* = 2 and *p* = 1 in Eq. 6), which promotes sparser traction fields [21], as the ones expected for TFM experiments. Unfortunately, it is not possible to express the solution in a closed form, thus requiring an optimization algorithm. Here, the Iterative Reweighted Least Squares (IRLS) algorithm [22] is used to solve the convex optimization problem and estimate ${\widehat{\mathbf{t}}}_{{\mathrm{L}}_1},$ as was used in [16, 18]. This algorithm approximates the L_1_-norm by a weighted L_2_-norm problem, updating those weights iteratively until convergence is reached. Then, at each iteration *s*:

12 $$ {\widehat{\mathbf{t}}}_{{\mathrm{L}}_1}^s={\left({\mathbf{K}}^{\mathrm{T}}\mathbf{K}+{\lambda}_{L_1}{\mathbf{W}}^s\right)}^{-1}{\mathbf{K}}^{\mathrm{T}}\mathbf{u} $$

where **W** ^*s*^ is the weight matrix at the *s* -th iteration. We considered that convergence was reached when the absolute value of the difference between the tractions estimated in two consecutive iterations was smaller than 10^−6^.

#### Full L_1_-Regularization in the Spatial Domain

The alternative, we propose in this manuscript, is to use the L_1_-norm for both data fidelity and penalty terms (*p* = *q* = 1 in Eq. 6). Analogously to the simple L_1_-regularization, there is no a closed form solution for the convex optimization problem and the IRLS algorithm can be used to estimate the cellular tractions. Then, the solution at each iteration *s* is given by:

13 $$ {\widehat{\mathbf{t}}}_{\mathrm{full}-{\mathrm{L}}_1}^s={\left({\mathbf{K}}^{\mathrm{T}}{\boldsymbol{W}}_{\boldsymbol{d}}^{\boldsymbol{s}}\mathbf{K}+{\lambda}_{\mathrm{full}-{\mathrm{L}}_1}{\mathbf{W}}^s\right)}^{-1}{\mathbf{K}}^{\mathrm{T}}{\boldsymbol{W}}_{\boldsymbol{d}}^{\boldsymbol{s}}\mathbf{u} $$

where ${\mathbf{W}}_d^s$ is the weight matrix of the data fidelity term at the *s*-th iteration.

### Stiffness matrix reduction

The main issue when working in the spatial domain with full-size real images and full-resolution displacements is the high computational requirements. For instance, for a typical field of view of 800 ∙ 800 pixels and storing each element of the stiffness matrix with double precision, its size in memory would be as large as is 2*N* ∙ 2*M* ∙ 8*Bytes* ~ 13 Terabytes, where *N* = *M* = 800 ∙ 800, being N the positions of the displacements used for the tractions estimation and M, the positions where the tractions would be recovered.

**Fig. 2:**
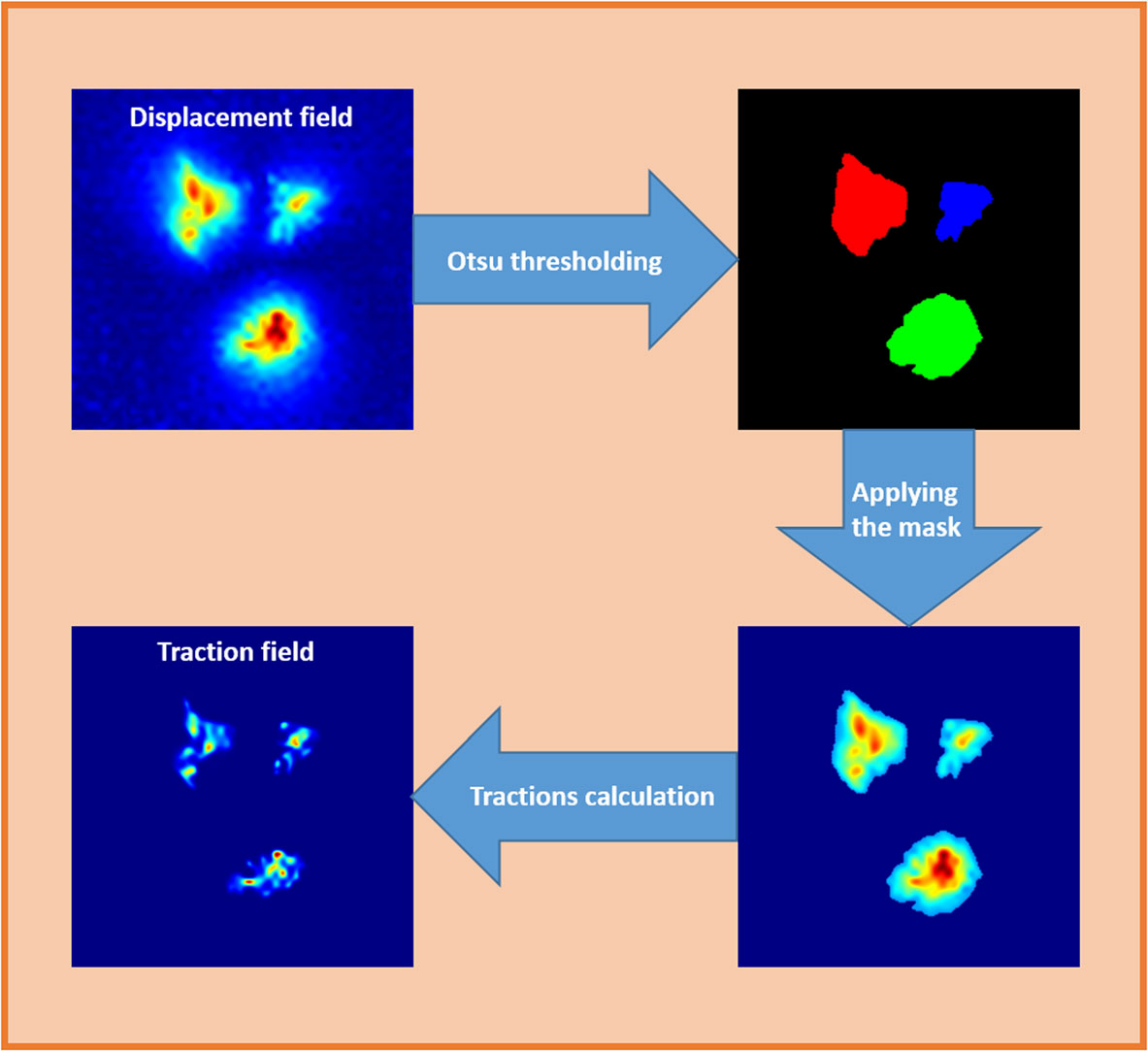
Main steps in Stiffness Matrix Reduction method. Areas with high displacement magnitude are segmented using an Otsu’s thresholding. After that, only the segmented displacements are used to estimate the traction field in every disjoint region

Consequently, solving the regularization problem in the spatial domain is not feasible on a standard desktop computer. To avoid this limitation and be able to work with both full-size real images and full-resolution displacements, we perform a reduction of the stiffness matrix by including a priori information inherent to TFM experiments (see Fig. 2). In particular, it is known that adherent cells exert tractions at discrete locations; namely, the focal adhesions. Thus, it is reasonable to assume that cellular tractions should be only estimated in certain regions, outside of which they should be zero. Note that given the conditions of the experiments we performed, we are not able to detect individual focal adhesions, but clusters of them as determined by cytoskeletal fibers. We refer to this approximation as stress footprints throughout the manuscript.

First, we consider that the regions presenting large displacements correspond to the sites of traction application, which is a priori a sufficient approximation, as the support of the traction field is much reduced than the one of displacement field. Hence, we can limit the number of locations where the tractions will be recovered (M) to the points inside these regions.

Furthermore, the signal-to-noise ratio of the displacements decreases with its magnitude and their information content quickly vanishes with the distance to the locations where tractions are exerted. Therefore, it is also reasonable to limit the locations of the measured displacements that will be used in the traction recovery (N) to those ones inside the segmented regions, as those outside would mainly contribute with noise in the system of equations.

To segment these regions, we apply an Otsu’s thresholding to the magnitude of the estimated displacement field. On our hands, the segmentation of the displacements extract disconnected regions of reduced size. Thus, the tractions in each of the disjoint regions can be recovered using only the information from the displacements located inside it. Consequently, the global stiffness matrix can be split in as many local stiffness matrices (with a drastically reduced size) as detected regions of interest, making the traction estimation feasible on a standard PC. Once all the local TFM problems have been solved, the contributions of each segmented region are combined together to maintain the original field size.

All the methods described in this section have been implemented in Matlab (MathWorks Inc.) code.

### Synthetic data

#### Simulated data generation

**Fig. 3:**
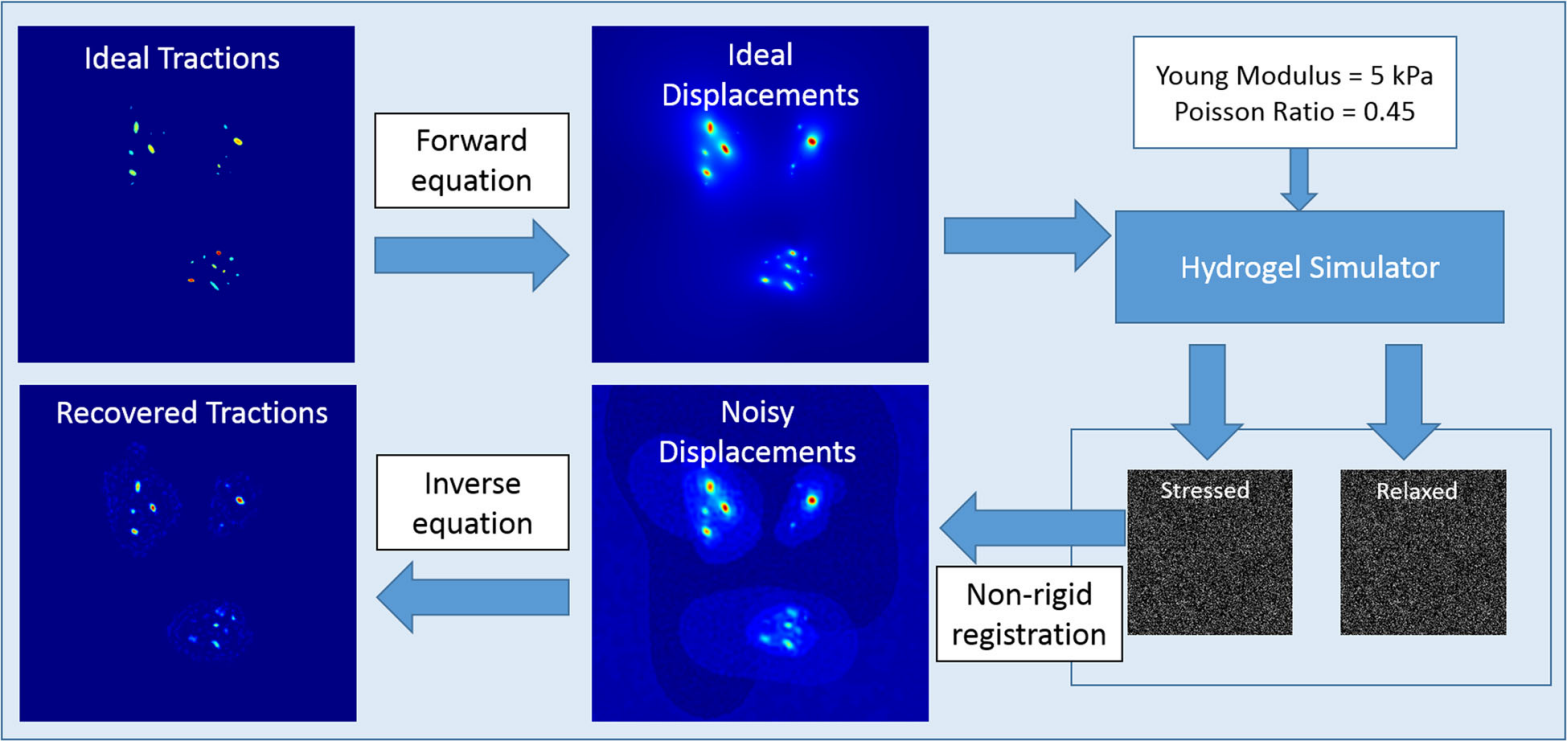
Pipeline of the synthetic data generation. From the ideal tractions, using the forward equation, the ideal displacements are computed. The simulator generates a synthetic relaxed hydrogel volume with fluorescent beads randomly distributed inside. The beads are distorted simulating the acquisition on a confocal microscope and the volume, contaminated by the characteristic noise. Based on the biomechanic properties selected for the hydrogel (namely, Young modulus and Poisson ratio), the stressed hydrogel is generated. Finally, using the stressed and relaxed hydrogels the inverse problem is solved using any of the presented approaches and tractions are recovered and compared with the ideal

To assess the performance of the different regularization schemes, we used our TFM simulator [23] to generate images of relaxed and stressed hydrogel containing embedded fluorescent beads as acquired by an optical microscope (see Fig. 3). Briefly, a simulated traction field was used to generate the displacements (Eq. 3) that move a large number of randomly distributed beads in the relaxed hydrogel to their new locations in the stressed hydrogel. Furthermore, the images containing the beads were generated taking into account the noise and the point spread function associated blurring that is introduced by the optical system and thus, represent a realistic synthetic dataset.

Then, the B-spline based non-rigid registration algorithm [12] was used to calculate a full-resolution displacement field from these images, as done in real TFM experiments. Lastly, the estimated displacements were fed to the regularized traction reconstruction algorithms under evaluation and the recovered tractions were compared to the ground-truth given by the simulated fields.

To create a realistic map of tractions that can be used with our TFM simulator, we used the tractions recovered from TFM experiments with real cells. Specifically, real traction fields computed with full L_1_-regularization were segmented using an extension of the classical isodata algorithm [24] that automatically selects five thresholds using a clustering approach. Then, we perform a binary segmentation based on the most restrictive of them to be used as the synthetic stress footprints mask.

Then, following the pipeline explained before, ten different realizations have been created for each simulated case, changing in each realization the position of the fluorescent beads. The selection of the optimal parameter λ for each regularization method has been performed by an exhaustive search. Namely, the inverse problem has been sequentially solved on a synthetic image randomly selected using a regularization parameter λ on the range [0,1] with a step size Δλ of 0.01. The regularization parameter λ used on the simulations for the evaluation of the algorithms was the one minimizing the cost functional defined on Eq. 6.

#### Error metrics

We have evaluated the error in the recovered traction field within a region of interest defined by the corresponding stress footprint. These footprints were obtained by segmenting the magnitude of the recovered tractions using an Isodata thresholding of two levels. These metrics are evaluated only in the recovered stress footprint areas.

Being ***t***_***gt***_ the simulated ground-truth traction field, $\widehat{\mathbf{t}}$ the retrieved traction field, and P and Q the total number of points within the respective stress footprints, we have defined the following error metrics:

##### Error in magnitude

Absolute error in the recovered traction magnitude (in percentage) defined as

14 $$ {e}_m=100\bullet {\sum}_{n=1}^{\widehat{n}}\left(\frac{\sum_{i=1}^{P_n}\left|\ \left\Vert {\widehat{\boldsymbol{t}}}_i\right\Vert -\left\Vert {\boldsymbol{t}}_{gt_i}\right\Vert\ \right|}{\sum_{\boldsymbol{i}=1}^{P_n}\left\Vert {\boldsymbol{t}}_{gt_i}\right\Vert}\right) $$

where $\widehat{n}$ is the number of recovered stress footprints, and *P*_*n*_ the points of the simulated stress footprint associated to each recovered footprint.

##### Error in angle

Average angular error (in percentage) in the recovered stress footprint, weighted at each point by the traction magnitude. It is normalized by the maximum error in degrees (180°) to give the result in percentage as given by

15 $$ {e}_{\mathrm{a}}=100\bullet {\sum}_{n=1}^{\widehat{n}}\left(\frac{\sum \limits_{i=1}^{P_n}\left(\frac{\left\Vert {\widehat{\boldsymbol{t}}}_i\right\Vert }{\sum \limits_{m=1}^{P_n}\left\Vert {\widehat{\boldsymbol{t}}}_m\right\Vert}\right)\left|{cos}^{-1}\left(\frac{{\widehat{\boldsymbol{t}}}_i\bullet {\boldsymbol{t}}_{gt_i}}{\left\Vert {\widehat{\boldsymbol{t}}}_i\right\Vert \bullet \left\Vert {\boldsymbol{t}}_{gt_i}\right\Vert}\right)\right|}{180}\right) $$

##### Error in area

Absolute error of the recovered stress footprint area (in percentage) as given by

16 $$ {e}_A=100\bullet {\sum}_{n=1}^{\widehat{n}}\left|\frac{\widehat{A}-{A}_{gt}}{A_{gt}}\right| $$

with $\widehat{A}$ and *A*_*gt*_ being the area of the recovered and ground-truth footprints, respectively.

##### Loss ratio of stress footprint areas

It is the ratio (in percentage) between stress footprints that the regularized TFM methods cannot recover (the number of false negatives) and the total number of simulated stress footprints synthetized by cell. It is defined as

17 $$ LFA=100\bullet \frac{n_{gt}-\widehat{n}}{n_{gt}} $$

where $\widehat{n}$ and *n*_*gt*_ are the number of recovered and ground-truth stress footprints.

##### Smallest detectable stress footprint areas

It is the smallest product between the area (in *μm* ^2^) and the peak magnitude (in kPa) of the recovered stress footprint areas. This metric reflects the dependence between the size and the magnitude of the stress footprints and it is defined as

18 $$ SFA=\underset{i}{\min \limits}\left({A_{gt}^i}^{\ast }{t}_{max}^i\right)\ (nN) $$

where ${A}_{gt}^i$ and ${t}_{max}^i$ are the ideal area and maximum traction amplitude of a recovered stress footprint i.

##### Statistical analysis

The statistical significance among different realizations and regularization schemes was evaluated for each error metric using the t-Student test with signrank Matlab function. A *p*-value smaller than 0.01 was considered to give statistical significance.

### Real data

We have used the presented algorithms to quantify the tractions exerted by Chinese Hamster Ovary (CHO-K1, American Type Culture Collection (ATCC; Rockville, MD), CCL 61) cells expressing Lifeact-GFP. These cells were cultured on a ~ 90 μm thick polyacrylamide hydrogel with a Young’s modulus of 5 kPa and a Poisson ratio of 0.45, containing embedded fluorescent polystyrene beads (0.2 μm in diameter, 647 nm of emission wavelength, ~1 bead per μm^2^). Images of the cells were acquired at multiple locations of the hydrogel surface with a 60× glycerol objective (NA = 1.3) mounted in a Leica SP5 Laser Scanning Confocal microscope with 0.32 μm of resolution in each direction of the plane. Time-lapse images of the stressed hydrogel were taken every 20 s during 25 min. An image of the relaxed hydrogel was taken after removing the cells.

## Results

### Synthetic data

To build a realistic synthetic dataset we used our TFM simulator with data from ten different real TFM experiments (i.e., cells) as explained in the previous section. A wide variety of test conditions were considered, specifically, the number of stress footprints per simulated case ranged between 10 and 120, their peak magnitude between 0.6 kPa and 4 kPa and their area between 0.25 μm^2^ and 20 μm^2^. The parameters to simulate the mechanics of the hydrogel and the acquisition of the bead images were chosen to be the same than those of the real experiments and are given below. Furthermore, multiple (10) realizations were simulated for each synthetic case to take into account the variability introduced by the random distribution of the beads inside the hydrogels. Finally, for the sake of comparison, the parameters controlling the amount of regularization were fixed for all the set-ups to ${\lambda}_{L_2}=0.2$, ${\lambda}_{L_1}=0.08$, ${\lambda}_{\mathrm{full}-{\mathrm{L}}_1}=0.1$. These values were independently determined for each regularization scheme after an exhaustive search minimizing the least square error between recovered and simulated traction fields. Additional file 1 shows an illustration of the changes in the recovered traction fields with the regularization parameter for each method.

**Fig. 4:**
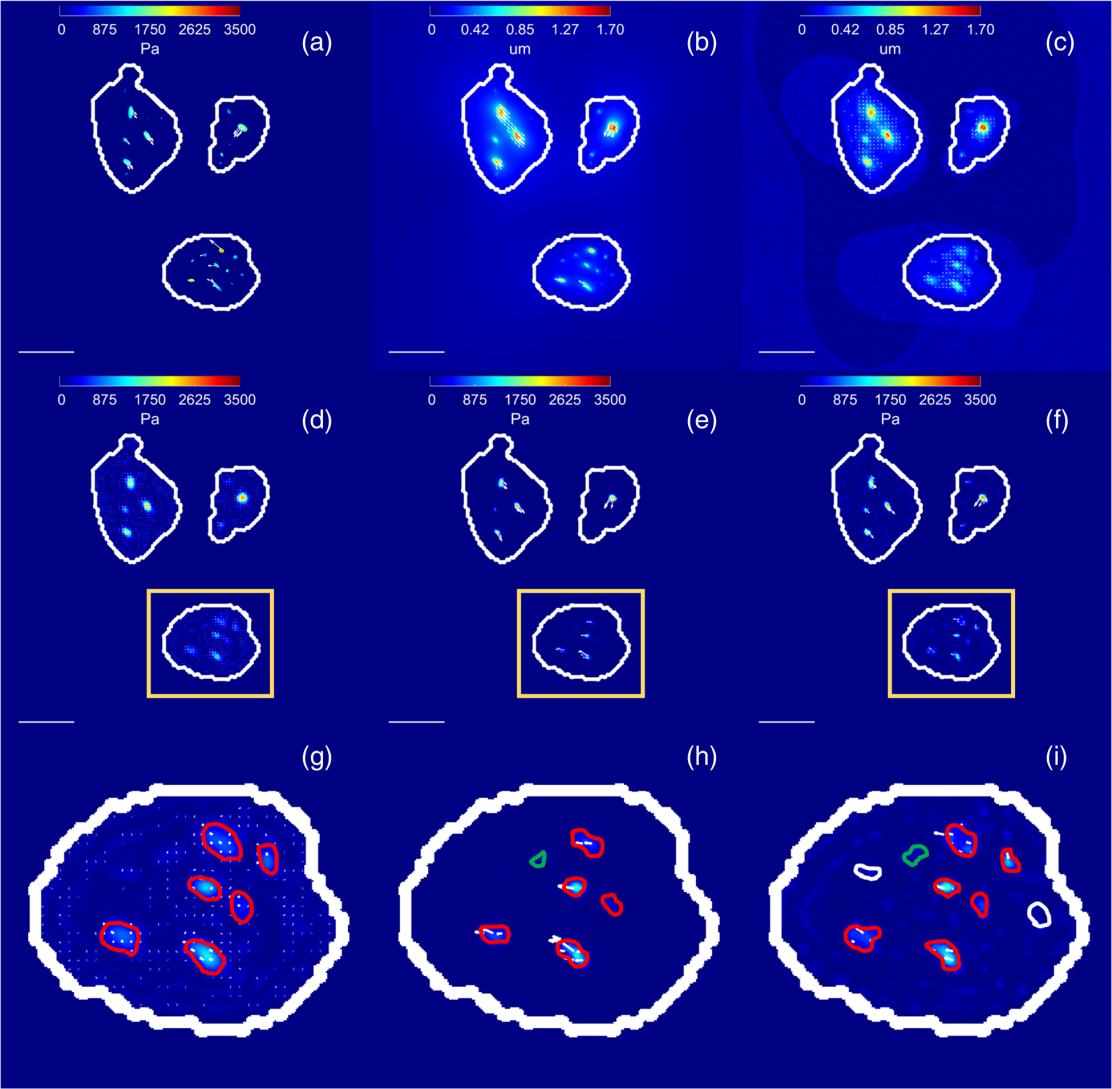
Example of traction recovery on a simulated cell. **a** Magnitude (in Pa) and direction (*arrows*) of simulated traction map; Magnitude (in μm) and direction (*arrows*) of ideal displacements **b** and noisy displacements **c**; recovered traction magnitude (in Pa) and direction (*arrows*) using: **d** Tikhonov regularization; **e** L_1_-norm regularization; **f** full L_1_-norm regularization and a zoom of the region of interest marked with a *yellow box* for **g** Tikhonov regularization; **h** L_1_-norm regularization; **i** full L_1_-norm regularization. The stress footprints recovered by the Tikhonov regularization are shown in *red*; These stress footprints when recovered with L_1_-norm and full L_1_-norm regularization are also shown in *red*; The additional stress footprint recovered by the L_1_-norm regularization is shown in *green*; Note that the rightmost top stress footprint detected by the Tikhonov regularization has disappeared; Lastly, the additional footprints recovered by the full L_1_-norm regularization are shown in *white*. The outline of the mask used for traction recovery is shown in *white*. The scale bar represents 30 μm

Figure 4 shows a representative example of simulated traction field (Fig. 4a) and ideal displacement field (Fig. 4b). The noisy displacements computed from the synthetic images of relaxed and stressed hydrogel are shown in Fig. 4c. Their corresponding tractions recovered by each regularization scheme are shown in Fig. 4d-f. The mask defining the regions were tractions are estimated is overlaid. As expected, a visual inspection of Fig. 4 reveals that L_1_-regularizations give sparser traction fields with less background signal and better estimation of traction magnitudes than the classical L_2_-norm based Tikhonov regularization. Furthermore, full L_1_-regularization is able to recover more stress footprints than the other methods.

**Fig. 5:**
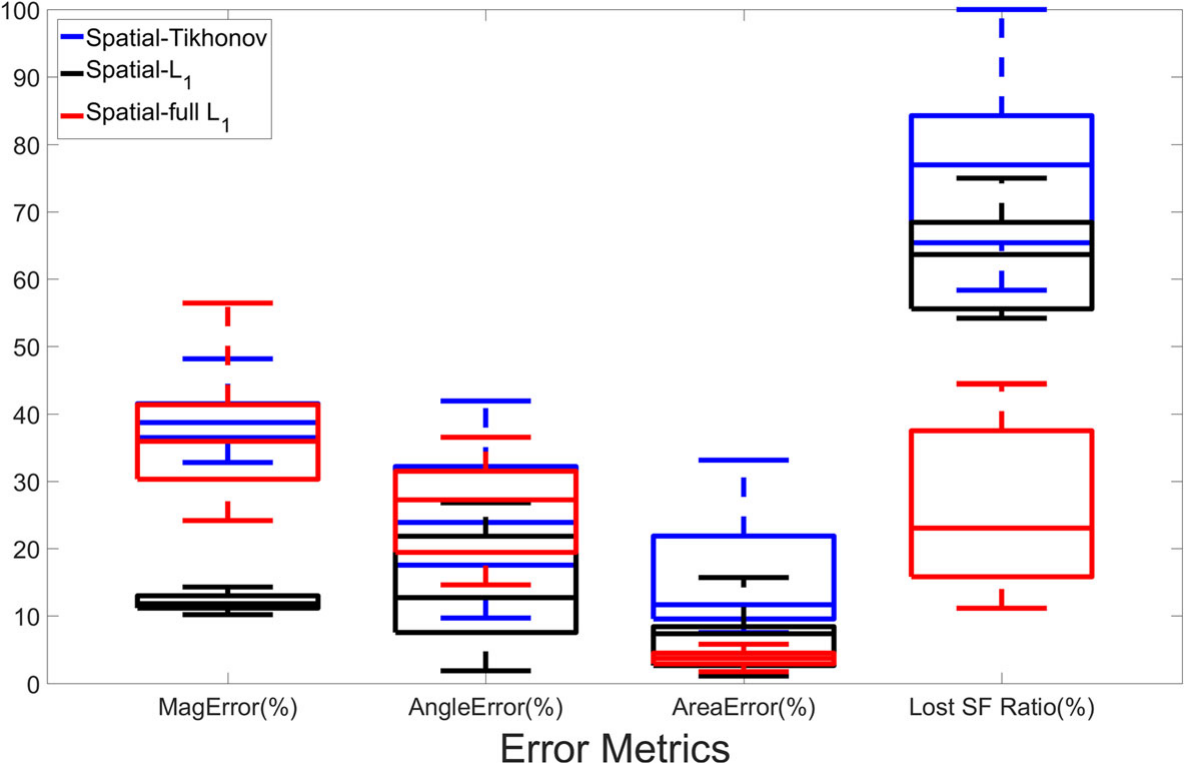
Quantitative comparison between different regularization schemes. Ten different traction maps have been considered and ten realizations for each have been computed. The Student’s t-test was used to compare the error measures computed by the different regularization techniques. All the differences were statistically significant (*p* < 0.001), except Angle Error between Tikhonov and full L_1_-regularization (*p* = 0.5)

**Fig. 6:**
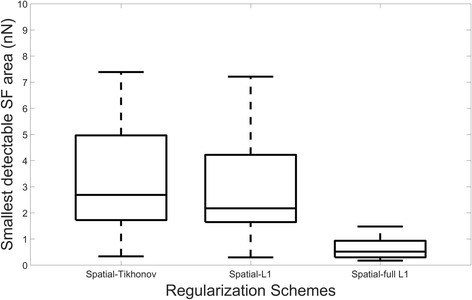
Comparison of the smallest detectable stress footprint areas (*nN*) for different regularization schemes. Ten different traction maps have been considered and ten realizations have been computed for each of them. All the differences are statistically significant (Student’s t-test, *p* < 0.005), except between Tikhonov and L_1_-regularization (*p* = 0.5)

Those results are quantitatively confirmed in Fig. 5 and Additional file 2. We observe how L_1_-regularization presents smaller errors in magnitude and angle of the recovered stress footprint areas than Tikhonov regularization and full L_1_-regularization, giving around three times smaller magnitude error (12%) than the other methods. Regarding the area error of the recovered stress footprints, full L_1_-regularization shows the best performance with an average error less than 4%, while L_1_-regularization has about 7% of error and Tikhonov present the worst performance with an average area error about 16%. Looking at the ratio of the lost stress footprints, full L_1_-regularization is able to recover more than the double of stress footprint areas (around 75%) than the other regularization methods (around 30% of them). In addition, in Fig. 6, it can be seen how full L_1_-regularization has the smallest detectable stress footprint area values. Namely, this method detects smaller stress footprint areas with reduced traction magnitude than the other methods. In particular, the smallest stress footprint areas detected in all experiments for each method are (Peak Magnitude (kPa), Area (*μm* ^2^), *mFA*(*nN*)): L_2_-regularization (0.89, 0.38, 0.33); L_1_-regularization (0.94, 0.31, 0.29); full L_1_-regularization (0.68, 0.25, 0.17).

Moreover, most of the differences between the error measures given for the different regularization schemes are statistically significant (*p* < 0.001). The exceptions are: The error in magnitude between Tikhonov and full L_1_-regularization and the smallest footprint detected between Tikhonov and L_1_-regularization.

### Real data

**Fig. 7:**
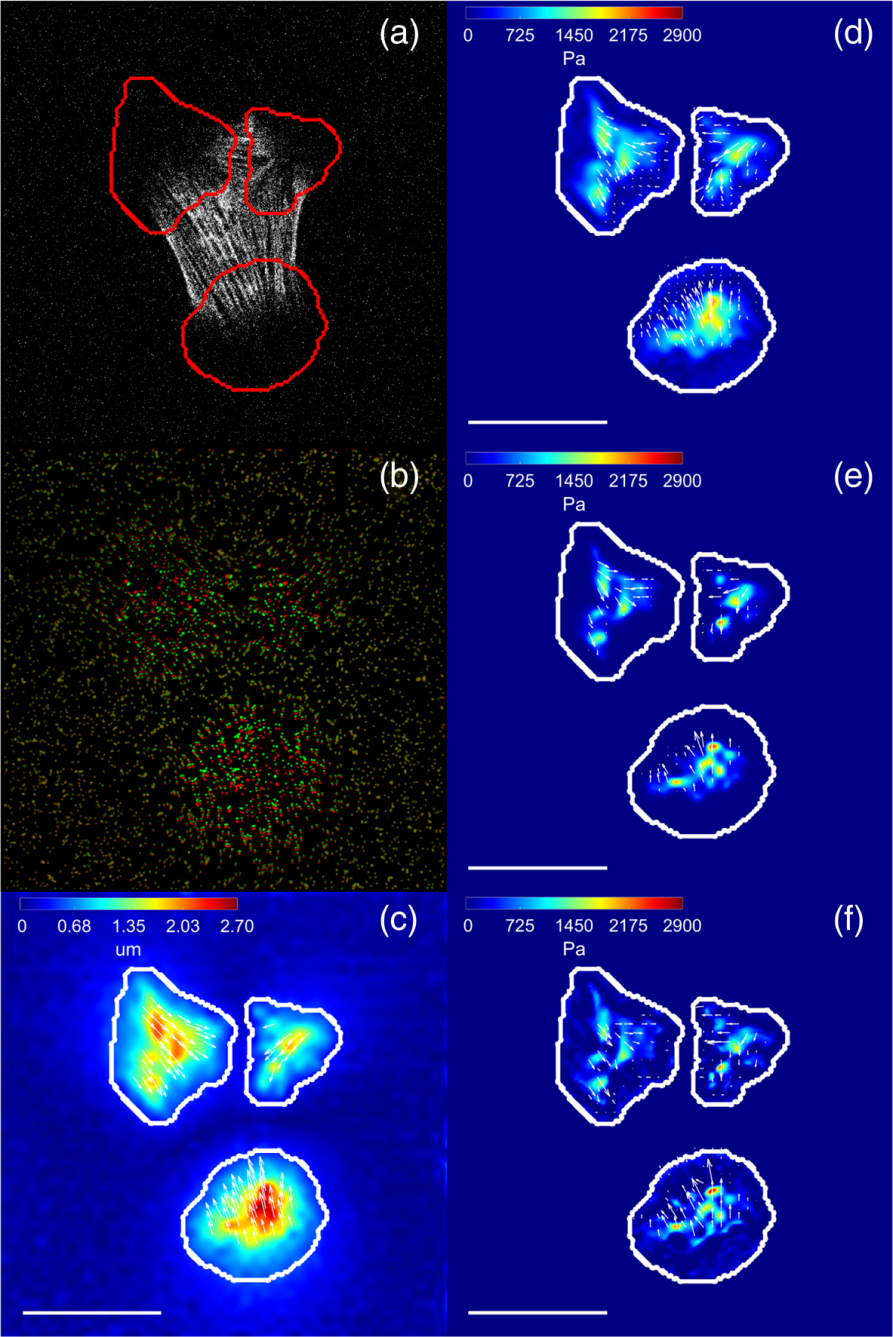
Example of traction recovery on a real cell. **a** CHO cell expressing Lifeact-GFP; **b** Pseudo-color image showing the fluorescent beads at the hydrogel surface. The beads of the unstressed and stressed hydrogels have been superposed and pseudo-colored in *red* and *green*, respectively; therefore, beads are colored *yellow* when not displaced. The contrast of the pseudo-color image has been modified to highlight the areas with bead displacements; **c** Magnitude (in μm) and direction (*arrows*) of in-plane displacements estimated from the bead images. Recovered traction magnitude (in Pa) and direction (*arrows*) using: **d** Tikhonov regularization; **e** L_1_-norm regularization; **f** full L_1_-norm regularization. The outline of the mask used for traction recovery is shown in *red* (**a**) and *white* (**c-f**). The *scale bar* represents 30 μm

In Fig. 7, an example of traction recovery on one frame of a real cell is shown. In Additional file 3, ten samples frames are presented. It can be seen how the results are qualitatively similar to those obtained for synthetic data.

Image of the cell (Fig. 7a), images of the stressed and relaxed polyacrylamide hydrogels (Fig. 7b) and the displacement estimation (Fig. 7c) are shown with the mask used for the tractions calculation, overlaid. In Fig. 7d-f, the recovered traction fields for each of the regularization schemes are shown. The Tikhonov regularization clearly smoothens the solution and introduces more background noise than L_1_-regularizations. Moreover, full-L_1_-regularization enables to distinguish between closer stress footprints as smaller stress footprints are recovered.

## Discussion

The use of the full L_1_-norm was previously introduced [25] and has been applied in different research areas, including biomedical applications [26–28], image processing [29, 30] and computer vision [31]. Whereas L_2_-data fidelity is useful for fields contaminated with Gaussian noise, microscopy images often contain more complex noise (mixed Poisson and Gaussian noise) as well as outliers. In these cases, applying the L_1_-norm data fidelity constraint is more appropriate.

Moreover, there has been previous theoretical work exploring the consequences of the use of the L_1_ norm in the data fidelity for image reconstruction [30]. In particular, it has been shown that some undesirable characteristics derived from the minimization of the absolute error instead of the least-squares error such as the lack of uniqueness of solutions, and the lack of continuous dependence on data, can be real assets. A major advantage of the L_1_ fidelity based model over the standard one is that the regularization imposed on solutions is more geometric, in the sense that the regularization process has less dependence on the contrast of image features than on their shapes; As distinct from the standard model, small features in the images maintain their contrast. In addition, L_1_ data fidelity works better reducing some artifacts like ringing and the presence of outliers [25, 29].

In Additional file 1, a comparison between the data fidelity and penalty term recovered by the regularization methods for a range of lambda values (from 0 to 0.22 with a step of 0.02) is shown. It is clear that full L_1_-regularization reduces the data fidelity term. This means that the matching between the estimated displacements and the inferred ones (the product of the stiffness matrix and calculated traction field) is greatly improved with respect to the other regularization schemes. This could be responsible for the strong decrease in the loss stress footprints ratio and it is the source of differences between the L_1_-regularizations.

Our results reveal that L_1_-regularizations give an improved spatial resolution (more important for full L_1_-regularization) in terms of sensitivity to detect smaller stress footprints in both synthetic and real data with respect to the classical zero-order Tikhonov regularization.

In our hands, L_1_-regularization of the penalty term presents smaller errors in magnitude and angle of the recovered stress footprint areas than the other regularization methods. It was expected that L_1_-regularization of the penalty term over-performed the results obtained by Tikhonov regularization as was already demonstrated in previous publications [16, 18]. Nevertheless, it was a surprising result for full L_1_-regularization and it is maybe due to our error metric evaluation procedure. Namely, we compute the error metrics over all the recovered stress footprints being the number recovered by full L_1_-regularization much larger. Even more, the ones recovered only by full L_1_-regularization tend to be the smaller in magnitude and area. Those risk to be the ones over which the reconstruction errors could be larger penalizing the computation of the error metrics.

**Fig. 8:**
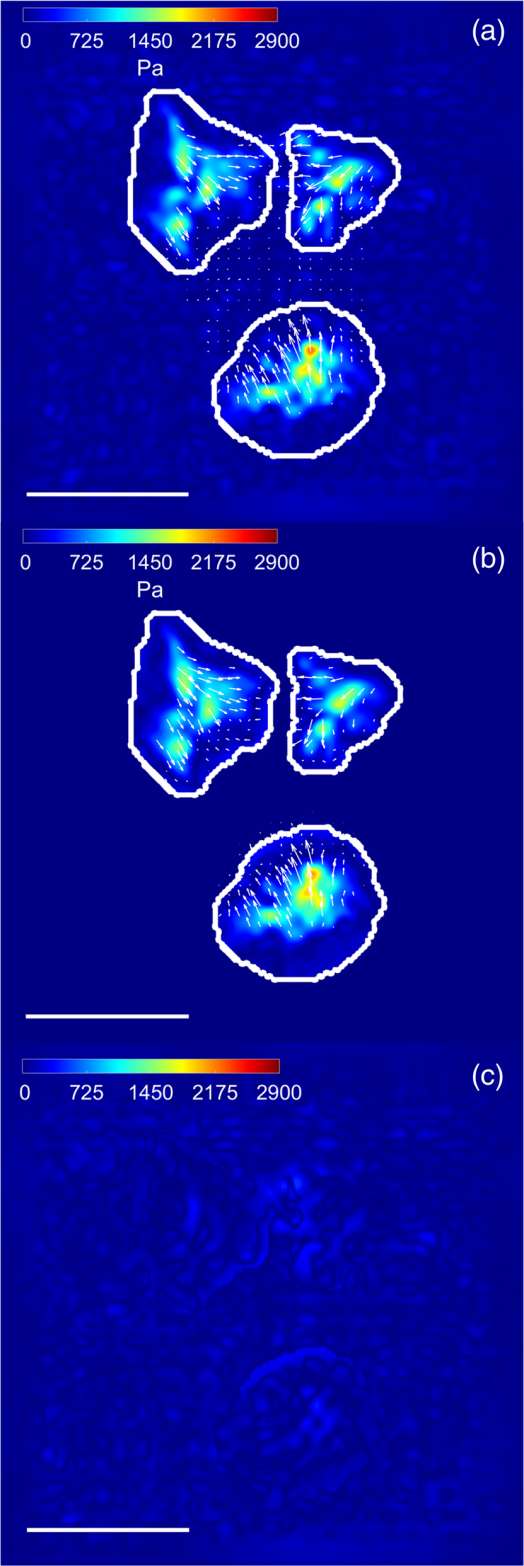
Comparison between spatial and Fourier Domain. Traction field magnitude (in Pa) and direction (*arrows*) from a real cell using L_2_-regularization: **a** Spatial domain; **b** Fourier domain. The outline of the mask used for traction recovery in the spatial domain is shown in *white*. **c** Absolute value of the difference between recovered tractions in (**a**) and (**b**). The *scale bar* represents 30 μm

As detailed in the previous sections, working in the spatial domain implies a high computational demand in terms of RAM and CPU usage due to the size of the stiffness matrix and the need to use an iterative optimization algorithm to recover the tractions, respectively. To overcome this severe problem, we have presented a method to reduce the size of the stiffness matrix that allows working with full-field microscopy images and full resolution displacement fields in the spatial domain. It is based on the calculation of forces in a few confined regions of interest defined with an Otsu thresholding over the magnitude of the estimated displacements. This approach results in a generous segmentation of the regions, guaranteeing no loss of information. Namely, more than 85% of the estimated displacements magnitudes are kept in all cases. This can be qualitatively observed in Fig. 8 for one of the synthetic cases. The stress footprints recovered using L_2_-regularization in the Fourier domain (using all data from the estimated displacement field) and in the spatial domain (using just the data covered by the mask) are visually similar, and no loss of information is apparent as shown in Fig. 8 (top) and (middle). Indeed, this approximation reduces the background signal but it does not cause loss of information of the recovered stress footprints (see Fig. 8 (bottom)). Moreover, there are not statistically significant differences between the quantitative error measures for both methods (t-test, *p* < 0.001) when computed on our synthetic dataset. As this solution is largely conservative, we believe that further refinements could reduce the computational demand even further. Finally, there exists an alternative TFM strategy called Traction Reconstruction with Point Forces (TRPF) [32] that also restricts the reconstruction of cellular tractions in the space to the focal adhesion sites at the cost of labeling them. Furthermore, in contrast to the method presented in this work, it would miss the tractions generated under the cellular nucleus in TFM setups considering transversal forces (known as 2.5D TFM setups) [33].

Recent publications have implemented novel methods incorporating relevant biomechanical constraints (i.e., the imposition of no forces outside the cell area and the assumption that if the cell is in equilibrium the sum of forces over the whole cell should be zero) [34, 35]. In our work, we have not imposed those constraints in the recovery of the tractions due, mainly, to two reasons: The first one is that in our real data, due to experimental limitations, the cell membrane might not be completely labeled. Consequently, it is not possible to accurately delineate the cell contour and, therefore, to impose that no forces are applied outside the cell. Regarding to the constraint of having the distribution of forces over the whole cell to sum up to zero, it has not been imposed in our experiments because in the stiffness matrix reduction approach the whole image is split in subregions to solve the traction recovery problem in small areas and, then, reduce the computational demand. This reduction implies solving the problem not in the whole cell and thus, it is not possible to add the force balance constraint. Nevertheless, for our real data, it has been verified that this sum is close to zero (less than 0.1% of the recovered maximum traction magnitude for all cases, see Additional file 4) (mean (Pa), standard deviation (Pa)): L_2_-regularization in Fourier Domain (−0.06, 0.24); L_2_-regularization in spatial domain (−1.26, 18.07); L_1_-regularization in spatial domain (−2.98, 18.59); full L_1_-regularization in spatial domain (−2.04, 17.88).

To reduce the computation time of the L_1_-regularization methods (full L_1_-regularization is about five times slower than L_1_-regularization and 20 times slower than L_2_-regularization in spatial domain), a more efficient implementation could be performed in a more suitable platform (i.e., using CUDA running over the FPGA), which would take advantage of every available resource of a desktop computer.

It is worth noting that we have simulated very small stress footprints in some of the synthetic traction maps. Those are very difficult to recover due to a variety of experimental constraints, namely, the random position of the beads, the limited microscope resolution and the possible proximity of other stress footprints with higher size or magnitude. A limitation of our experimental set-up is the fact that we use relatively large beads (200 nm) restraining the density of fiducial markers embedded in the hydrogel to avoid clustering. This issue could be partly solved by using smaller beads (~40 nm) that could be packed with a higher density and closely to the surface [16]. A recently proposed and elegant alternative is to use a polydimethylsiloxane (PDMS) silicone hydrogel with printed static quantum dots of 200 nm of diameter distributed regularly (with 1,5 μm of separation) [36].

As per the experiments with real data we have only performed a qualitative comparison of the different regularization methods. Our current work focuses on designing experiments in which the cellular focal adhesions will be stained in vivo. For this purpose, we are constructing cells stably expressing bona fide markers of focal adhesion coupled to fluorescent markers (e.g. EGFP), which are commonly used in these types of experiments [6]. Our motivation is to build ground truth data in which would be possible to establish quantitatively the resolution reached by the different regularization schemes.

We believe this improvement in sensitivity and spatial resolution could be relevant when combined with advanced microscopy methods, for cell biomechanics studies willing to drive the focus toward the characterization of individual focal adhesions instead of clusters of them as historically performed.

## Conclusion

In this manuscript, we have implemented full L_1_-regularization to recover cellular tractions in TFM experiments, showing its good performance compared with classical Tikhonov regularization and simple L_1_-regularization both in simulated and real data.

The main characteristic of full L_1_-regularization is the huge improvement in the sensitivity and the spatial resolution, which allows recovering smaller stress footprints compared with the state-of-the-art regularization methods. In addition, we have demonstrated the improved performance of L_1_-regularizations, giving smaller errors in the recovered traction field and resulting in less background noise than Tikhonov regularization.

In this work, we have also presented a method to make feasible the recovery of the tractions exerted by whole cells on full-field microscope images when working in the spatial domain. We have further demonstrated the suitability of the approach for the analysis of both realistic simulations and real data experiments.

## Additional files

Additional file 1: Comparison of the different terms involved in the cost functional for the different regularization methods and a swept of the regularization parameter (*λ*) values. The first row is for Tikhonov regularization, the second one for L_1_-regularization and the third row is for full L_1_-regularization. The columns from left to right show: traction field (in Pa), displacement field (in μm), ***K*** ∙ ***t*** in Eq. 6 (in μm) and data fidelity term (||**Kt** − **u**||_*q*_ in Eq. 6) (in μm). The outline of the mask used for traction recovery is shown in white. The scale bar represents 30 μm. (GIF 18689 kb)

Additional file 2: Quantitative results from synthetic data. Table with the different error metrics (mean±standard deviation) obtained by the different regularization methods on the synthetic data. Ten different traction maps have been considered and ten realizations for each one of them. The best results for each metric are highlighted in red. For all cases, the differences between the realizations are statistically significant (*p* < 0.001) as computed by a Student’s test. (TIFF 431 kb)

Additional file 3: Ten samples frames illustrating the whole TFM experiment and comparing the traction recovery with the different regularization methods. For each frame: (Top row, left) CHO cell expressing Lifeact-GFP; (Top row, center) Pseudo-color image showing the fluorescent beads at the hydrogel surface. The beads of the unstressed and stressed hydrogels have been superposed and pseudo-colored in red and green, respectively; therefore, beads are colored in yellow when not displaced. The contrast of the pseudo-color images has been modified to highlight the areas with bead displacements; (Top row, right) Magnitude (in μm) and direction (arrows) of in-plane displacements estimated from the bead images. (Bottom row) Recovered traction magnitude (in Pa) and direction (arrows) using: (Left) Tikhonov regularization; (Center) L_1_-norm regularization; (Right) full L_1_-norm regularization. The outline of the mask used for traction recovery is shown in red (Top row, right) and white (for the rest). The scale bar represents 30 μm. Frame #5 corresponds to Fig. 7 in the main manuscript. (GIF 10461 kb)

Additional file 4: Force balance over real cells. Table with the mean (in Pa and in percentage of the maximum traction magnitude) and the standard deviation (in Pa) of the sum of forces over the whole cell for each regularization scheme and for all real dataset. (TIFF 110 kb)

## Abbreviations

CHO-K1: Chinese Hamster Ovary
CUDA: Compute Unified Device Architecture
ECM: Extracellular Matrix
FA: Focal Adhesion
FPGA: Field Programmable Gate Array
GFP: Green Fluorescent Protein
IRLS: Iterative Reweighted Least Squares
LFA: Lost ratio of stress Footprint Areas
PDMS: PolyDiMethylSiloxane
PSF: Point Spread Function
SF: Stress Footprint
SFA: Smallest detectable stress Footprint Areas
TFM: Traction Force Microscopy
TRPF: Traction Reconstruction with Point Forces
