# Supplementary figures and images for: Full L1-regularized Traction Force Microscopy over whole cells
Source: BMC Bioinformatics. 2017 Aug 10;18:365. doi: 10.1186/s12859-017-1771-0 (PMC5550960; doi:10.1186/s12859-017-1771-0)

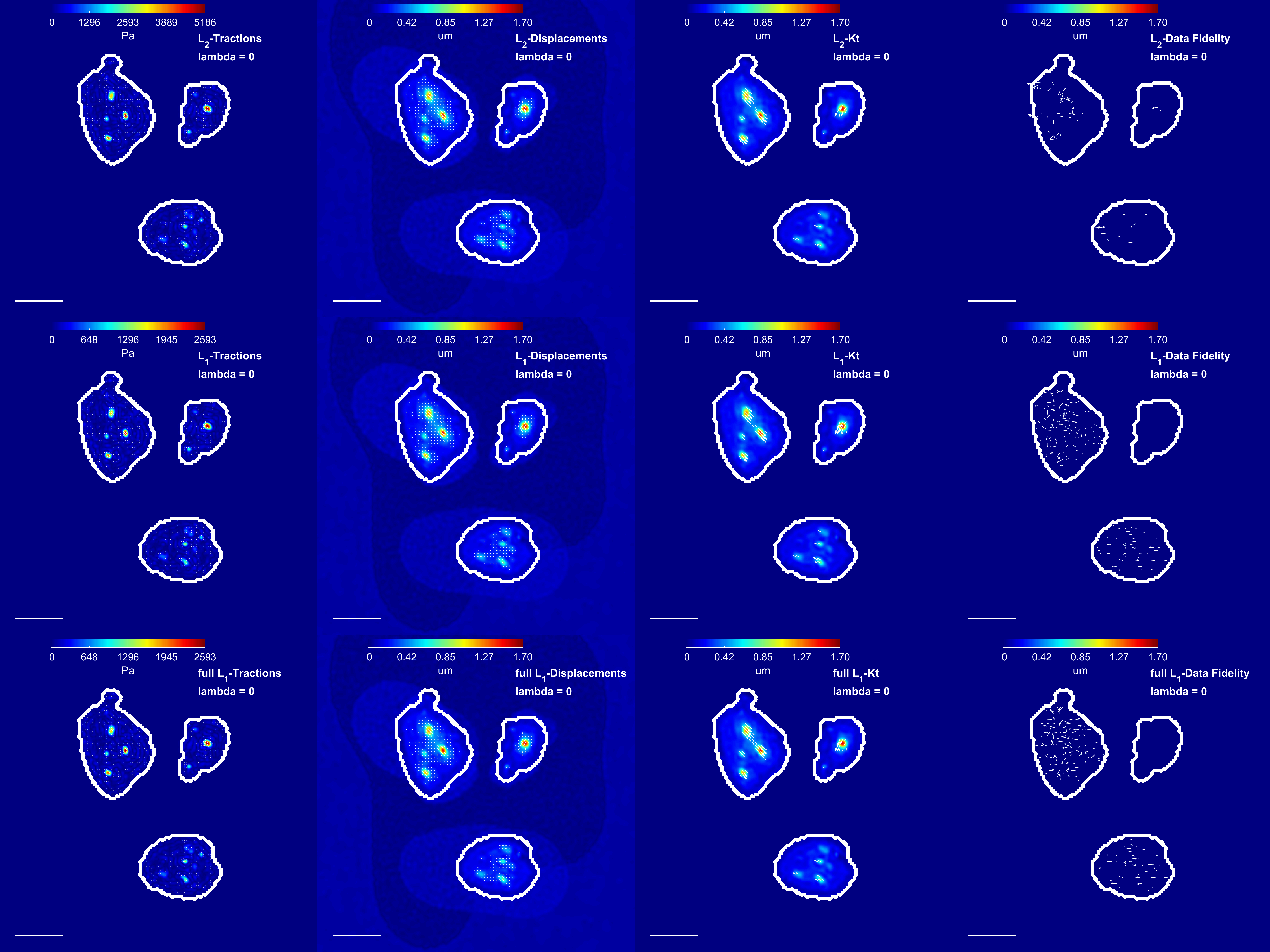

Supplement: Supplementary file 1 — Comparison of the different terms involved in the cost functional for the different regularization methods and a swept of the regularization parameter (λ) values. The first row is for Tikhonov regularization, the second one for L1-regularization and the third row is for full L1-regularization. The columns from left to right show: traction field (in Pa), displacement field (in μm), K ∙ t in Eq. 6 (in μm) and data fidelity term (||Kt − u||q in Eq. 6) (in μm). The outline of the mask used for traction recovery is shown in white. The scale bar represents 30 μm. (GIF 18689 kb) [file 12859_2017_1771_MOESM1_ESM.gif]

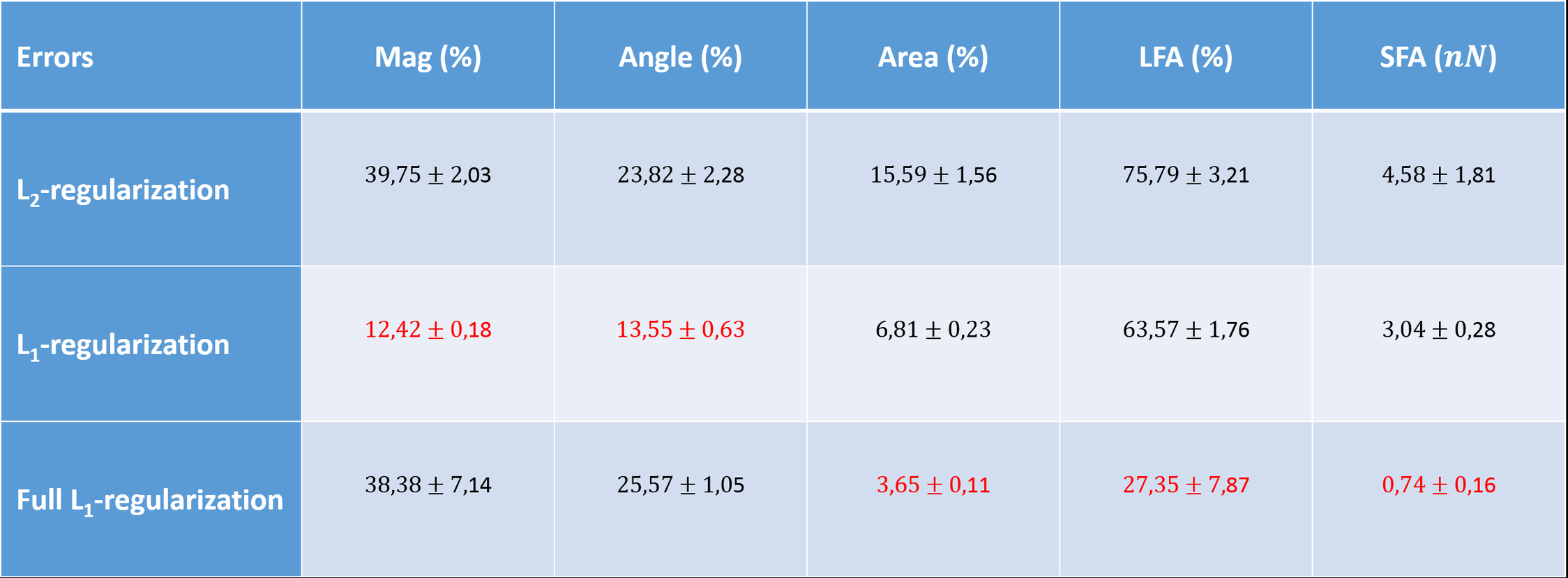

Supplement: Supplementary file 2 — Quantitative results from synthetic data. Table with the different error metrics (mean±standard deviation) obtained by the different regularization methods on the synthetic data. Ten different traction maps have been considered and ten realizations for each one of them. The best results for each metric are highlighted in red. For all cases, the differences between the realizations are statistically significant (p < 0.001) as computed by a Student’s test. (TIFF 431 kb) [file 12859_2017_1771_MOESM2_ESM.tif]

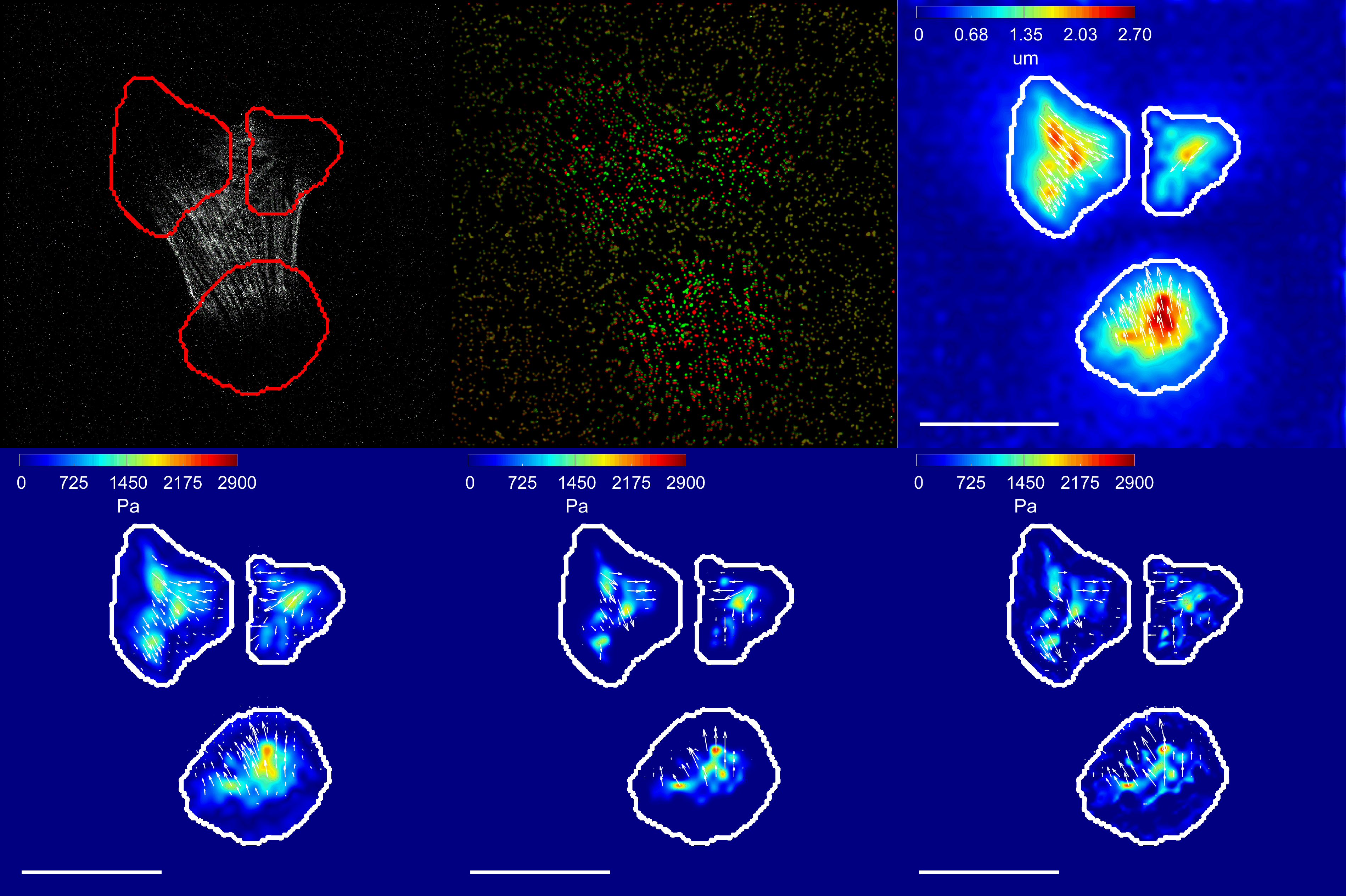

Supplement: Supplementary file 3 — Ten samples frames illustrating the whole TFM experiment and comparing the traction recovery with the different regularization methods. For each frame: (Top row, left) CHO cell expressing Lifeact-GFP; (Top row, center) Pseudo-color image showing the fluorescent beads at the hydrogel surface. The beads of the unstressed and stressed hydrogels have been superposed and pseudo-colored in red and green, respectively; therefore, beads are colored in yellow when not displaced. The contrast of the pseudo-color images has been modified to highlight the areas with bead displacements; (Top row, right) Magnitude (in μm) and direction (arrows) of in-plane displacements estimated from the bead images. (Bottom row) Recovered traction magnitude (in Pa) and direction (arrows) using: (Left) Tikhonov regularization; (Center) L1-norm regularization; (Right) full L1-norm regularization. The outline of the mask used for traction recovery is shown in red (Top row, right) and white (for the rest). The scale bar represents 30 μm. Frame #5 corresponds to Fig. 7 in the main manuscript. (GIF 10461 kb) [file 12859_2017_1771_MOESM3_ESM.gif]

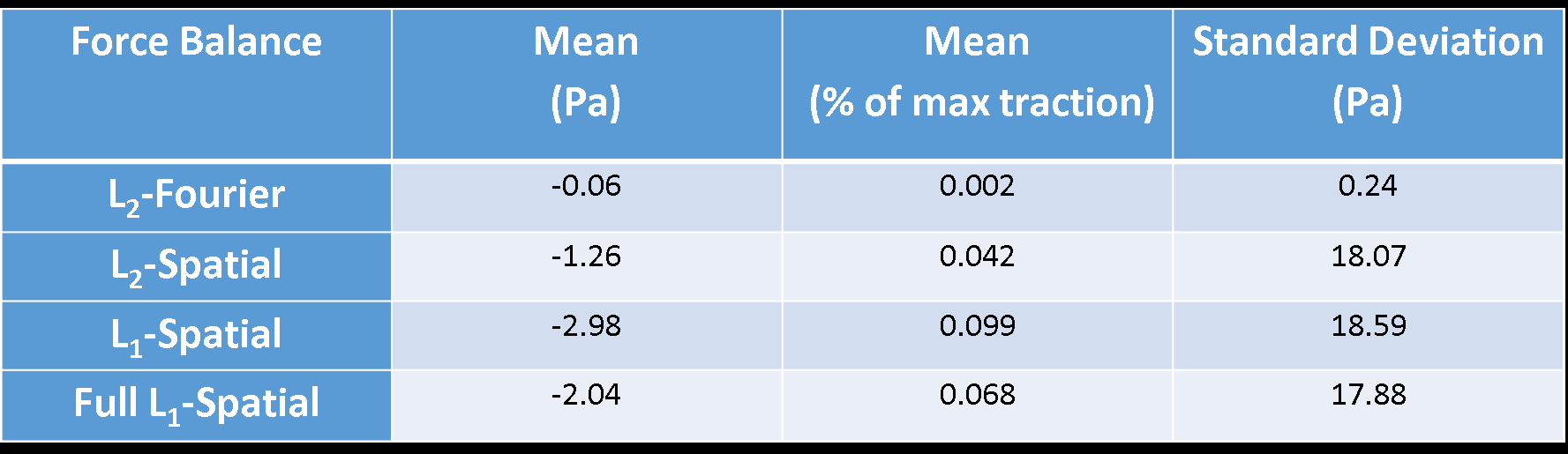

Supplement: Supplementary file 4 — Force balance over real cells. Table with the mean (in Pa and in percentage of the maximum traction magnitude) and the standard deviation (in Pa) of the sum of forces over the whole cell for each regularization scheme and for all real dataset. (TIFF 110 kb) [file 12859_2017_1771_MOESM4_ESM.tif]
